# Factor structure of the parental bonding instrument for pregnant Japanese women

**DOI:** 10.1038/s41598-022-22017-2

**Published:** 2022-11-09

**Authors:** Naoki Fukui, Yuichiro Watanabe, Koyo Hashijiri, Takaharu Motegi, Maki Ogawa, Jun Egawa, Takayuki Enomoto, Toshiyuki Someya

**Affiliations:** 1 Department of Psychiatry, Niigata University Graduate School of Medical and Dental Sciences, 757 Asahimachidori-Ichibancho, Chuo-Ku, Niigata, 951-8510 Japan; 2 Department of Obstetrics and Gynecology, Niigata University Graduate School of Medical and Dental Sciences, Niigata, Japan

**Keywords:** Physiology, Health care, Medical research

## Abstract

The parental bonding instrument (PBI) is often used to examine the perceptions of children and adolescents regarding parenting practices. Previous studies have investigated the factor structure of the PBI. However, although it is important to examine the relationships between the perceived parenting practices and perinatal mental health, few studies have included perinatal women. We aimed to accurately clarify which PBI factor structure was useful in assessing perinatal women (n = 4633). Furthermore, we evaluated the measurement invariance between primipara and multipara groups, and between the paternal and maternal PBI forms. Our exploratory and confirmatory factor analyses revealed that a three-factor PBI structure was most plausible for perinatal women. Moreover, we found complete invariance (residual invariance) of the PBI ratings across primipara and multipara women for the paternal and maternal forms. In contrast, we found weak invariance (metric invariance) of the PBI ratings across the paternal and maternal forms. Our participants tended to rate fathers as less caring and less overprotective than mothers. This three-factor structure shows measurement invariance in perinatal women and can be used to accurately determine how the perceived parenting style before adolescence influences women’s mental health in the perinatal period.

## Introduction

The parental bonding instrument (PBI) is often used in order to evaluate parenting style as experienced by older children and adults^1^. It have been reported that poor parenting styles evaluated with PBI such as “low care” and “high overprotection” are associated with depressive symptoms in perinatal women^2–4^. In our recent study with 1301 pregnant women, we used PBI, Mother-to-Infant Bonding Scale, and Hospital Anxiety and Depression Scale and reported that maternal–infant bonding was directly as well as indirectly affected via anxiety and depression by perceived negative parenting before adolescence^5^. Maternal–infant bonding impairment during the perinatal period is thought to lead to lack of or delayed emotional responses to the infant, irritability, hostility, aggressive impulses, or rejection of the child^6,7^. Consequently, using the PBI to reveal the parents’ perceptions of the mother’s parenting would be helpful to identify women at risk of maternal–infant bonding impairment in the perinatal period.

In our abovementioned recent study^5^, we adopted the two-dimensional model of “care” and “overprotection” designed by Parker et al.^1^. Previous studies performed to explore the factor structure of the PBI have produced inconsistent results. The PBI with a two-, three-, or four-factor structure has been used in the general population in several countries^8–18^. In studies that proposed the two-factor model, almost the same two-dimensional model of “care” and “overprotection” designed by Parker et al. have been adapted. In the three-factor model, “overprotection” has tended to be divided into two factors. In the four-factor model, each of “care” and “overprotection” has tended to be divided into two factors. As the factor structure of the PBI varies depending on the subjects and populations, it is necessary to examine the factor structure of each study. However, only one study has analyzed the PBI data of Japanese perinatal women^19^. The study of Japanese women collected PBI data from 932 primipara women at 25 weeks of gestation and at 1 month postpartum, with a three-factor structure of “Care,” “Indifference,” and “Autonomy”^19^; however, this previous study did not assess PBI data from multipara women and could not compare the PBI data between primipara and multipara women.

In many studies, data from the paternal and maternal PBI forms have been analyzed together. Furthermore, many studies have discussed the way that the mother’s and father’s parenting styles as evaluated by the PBI affect the psychological symptoms of their children. However, before making such assessments, it is important to perform multiple-group invariance analyses to determine the extent to which the derived factor structure is comparable between the paternal and maternal PBI forms.

The present study included a large number of perinatal women and analyzed the maternal and paternal PBI data separately. The aim of the present study was to accurately clarify the optimal factor structure of the PBI for a perinatal study. Furthermore, we evaluated the measurement invariance between primipara and multipara groups, and between the paternal and maternal PBI forms.

## Materials and methods

### Ethics statement

This study followed the principles of the Declaration of Helsinki and was approved by the Ethics Committee of Niigata University. Written informed consent was obtained from all participants.

### Participants

We have conducted the Perinatal Mental Health Research Project between March 2017 and December 2019^20–23^ and this study was part of the project. Our Japanese participants were recruited from 34 obstetric institutions in Niigata Prefecture, Japan. In our project, we collected the data of PBI at the time of entry of the study, and data of Hospital Anxiety and Depression Scale and Mother-to-Infant Bonding Scale at three time points: early pregnancy (approximately 12–15 weeks gestation), late pregnancy (approximately 30–34 weeks gestation) and postpartum (4 weeks after childbirth). We excluded pregnant women under 18 years of age and pregnant women with serious physical complications, serious pregnancy complications, or severe psychiatric disorders (e.g., severe schizophrenia and severe depression).

### Measurements

The PBI is self-administered questionnaire that retrospectively measures participants’ perceptions on perceived parenting by their parents before the age of 16 years^1^. The PBI is composed of two parts, the perceptions of perceived parenting by the mother and the father. Participants answer the two parts, each consisting of 25 items that rated on a four-point Likert-type scale from 0 to 3. This study adapted the Japanese version of the PBI^24^.

We collected data of obstetric factors: parity, type of conception (natural conception or others), number of fetuses (single or multiple pregnancy), gestational age at delivery (full term delivery or preterm delivery) and type of delivery (normal vaginal delivery or others).

### Statistical analysis

We randomly divided subjects with PBI data into two groups. Using the first group, we conducted an exploratory factor analysis (EFA) using the factor number determined by the Scree test and Promax rotation. We then created subscales consisting of items for which the loading on each factor was 0.30 or higher.

Using the second group, we performed a confirmatory factor analysis (CFA) using the optimal factor structure extracted from the EFA. Before performing the CFA, we excluded items with a factor loading of ≥ 0.30 for multiple factors in the EFA of either the maternal or paternal forms. We used the comparative fit index (CFI) and the root mean square error of approximation (RMSEA) to evaluate an acceptable fit (CFI ≥ 0.90 and RMSEA ≤ 0.08)^25^ between the models and the data.

Analyses of multiple-group invariance were performed to determine the extent to which the factor structure found in the CFA was comparable between primipara and multipara groups, and between the paternal and maternal PBI forms. Four levels of measurement invariance were sequentially evaluated (configural, metric, scalar, and residual invariance), where each level introduced more equality constraints across groups. If the ΔCFI was less than 0.01 even with one more constraint, it was considered that the measurement invariance would be maintained up to that level^26^.

T-tests were used to compare the mean value of each factor between the paternal and maternal forms, and between primipara and multipara women. The level of significance was set at p < 0.006, in accordance with the Bonferroni correction of nine statistical tests. Statistical analyses were performed using IBM SPSS Statistics version 25 (IBM Japan, Tokyo, Japan) and Amos version 25.0.0 (IBM Japan, Tokyo, Japan).

## Results

### Descriptive statistics

We collected the PBI data of 5053 perinatal women, and used the data with no missing values of 4633 perinatal women (mean age 31.6 ± 4.8 years) in our analyses. The number of subjects with 0, 1, 2 and 3 or more childbirth was 1955, 1521, 506 and 112, respectively. The number of subjects with natural conception and others was 3633 and 448, respectively. The number of subjects with single and multiple pregnancy was 4036 and 37, respectively. The number of subjects with full term delivery and preterm delivery was 3915 and 129, respectively. The number of subjects who had normal vaginal delivery and others was 3075 and 1021, respectively. The number of subjects with missing data on parity, type of conception, number of fetuses, gestational age at delivery and type of delivery was 539, 552, 560, 589 and 537, respectively.

### Exploratory factor analyses

We performed the EFA using the first group (n = 2303). In the EFA, the Scree test and Promax rotation showed that three was the most appropriate number of factors in both the paternal and maternal forms. Table 1 shows the EFA results of the paternal and maternal PBI data.

**Table 1:** Exploratory factor analyses of the paternal and maternal PBI data (n = 2303).

Items no. statement	Factor coefficient					
	Paternal form			Maternal form		
	Factor 1	Factor 2	Factor 3	Factor 1	Factor 2	Factor 3
1. Spoke to me with a warm and friendly voice	**0.808**	0.049	0.118	**0.799**	0.115	0.047
2. Did not help me as much as I needed	**− 0.502**	0.062	0.013	**− 0.469**	− 0.078	− 0.006
3. Let me do those things I liked doing	0.059	0.022	**0.691**	0.092	0.003	**0.721**
4. Seemed emotionally cold to me	**− 0.676**	− 0.009	0.083	**− 0.806**	0.052	0.100
5. Appeared to understand my problems and worries	**0.694**	0.026	− 0.039	**0.633**	0.021	0.152
6. Was affectionate to me	**0.707**	0.185	0.089	**0.780**	0.121	0.120
7. Liked me to make my own decisions	**0.328**	− 0.098	**0.343**	**0.303**	− 0.181	0.273
8. Did not want me to grow up	− 0.292	**0.377**	− 0.006	− 0.188	**0.320**	− 0.144
9. Tried to control everything I did	− 0.047	**0.466**	− 0.284	− 0.100	**0.560**	− 0.178
10. Invaded my privacy	− 0.139	**0.395**	− 0.194	− 0.202	**0.486**	− 0.137
11. Enjoyed talking things over with me	**0.863**	− 0.023	0.114	**0.837**	0.106	− 0.015
12. Frequently smiled at me	**0.885**	0.013	0.164	**0.896**	0.174	− 0.004
13. Tended to baby me	0.025	**0.601**	0.103	0.047	**0.712**	0.010
14. Did not seem to understand what I needed	**− 0.517**	− 0.089	**0.302**	**− 0.463**	0.278	− 0.166
15. Let me decide things for myself	0.080	− 0.101	**0.541**	0.083	− 0.217	**0.524**
16. Made me feel I wasn’t wanted	**− 0.397**	− 0.087	0.161	**− 0.634**	0.163	0.140
17. Could make me feel better when I was upset	**0.719**	− 0.022	0.081	**0.683**	0.032	0.062
18. Did not talk with me very much	**− 0.821**	0.171	− 0.049	**− 0.725**	0.019	0.147
19. Tried to make me dependent on her/him	0.129	**0.530**	0.129	0.094	**0.654**	0.099
20. Felt I could not look after myself unless	− 0.039	**0.541**	0.043	− 0.033	**0.789**	0.123
21. Gave me as much freedom as I wanted	− 0.082	0.075	**0.972**	− 0.112	0.031	**0.987**
22. Let me go out as often as I wanted	− 0.166	0.121	**0.846**	− 0.163	0.088	**0.929**
23. Was overprotective of me	**0.311**	**0.496**	− 0.064	0.285	**0.684**	− 0.034
24. Did not praise me	**− 0.733**	0.059	0.044	**− 0.716**	0.105	0.118
25. Let me dress in any way I pleased	0.140	0.057	**0.437**	0.220	− 0.010	**0.416**

Factor loadings of ≥ 0.30 are shown in bold.

### Confirmatory factor analyses

Before performing the CFA, three items (7, 14, and 23) that had a factor loading of 0.3 or higher for multiple factors in the EFA were excluded from the paternal form (Table 1). We also excluded three items (7, 14, and 23) from the maternal form in accordance with the criteria described in the “Materials and methods” section (Table 1). We performed the CFA using the second group (n = 2330). Table 2 shows the standardized coefficients between each item and factor in the CFA. The three-factor structure was confirmed to have an acceptable fit for both the paternal (CFI = 0.900, RMSEA = 0.070) and maternal PBI data (CFI = 0.906, RMSEA = 0.072).

**Table 2:** Confirmatory factor analysis of the three-factor model (n = 2330).

PBI item	Standardized coefficients	
	Paternal form	Maternal form
**Factor 1**		
1	0.796	0.789
2	0.488	0.410
4	0.708	0.724
5	0.710	0.745
6	0.789	0.818
11	0.802	0.790
12	0.834	0.830
16	0.483	0.602
17	0.695	0.720
18	0.724	0.683
24	0.711	0.680
**Factor 2**		
8	0.529	0.582
9	0.742	0.786
10	0.673	0.716
13	0.416	0.581
19	0.327	0.473
20	0.508	0.614
**Factor 3**		
3	0.747	0.796
15	0.692	0.710
21	0.861	0.868
22	0.675	0.706
25	0.498	0.551
Covariance between factor 1 and factor 2	− 0.461	− 0.542
Covariance between factor 2 and factor 3	0.749	0.715
Covariance between factor 1 and factor 3	− 0.576	− 0.583

### Comparison with other models

Table 3 shows the results of the CFA for factor models used in other Japanese studies with our second set of PBI data (n = 2330). Our three-factor structure induced by the abovementioned EFA and CFA was completely consistent with that reported by Sato et al.^19^ using the PBI data of Japanese perinatal women. We also evaluated the fit of our model in comparison with another two studies. In CFA using our paternal PBI data and the two-factor model of Kitamura et al.^24^, the fit indices were CFI = 0.844, RMSEA = 0.082. In CFA using our paternal PBI data and the four-factor model of Uji et al.^17^, the fit indices were CFI = 0.879, RMSEA = 0.073. In CFA using our maternal PBI data and the two-factor model of Kitamura et al.^24^, the fit indices were CFI = 0.834, RMSEA = 0.089. In CFA using our maternal PBI data and the four-factor model of Uji et al.^17^, the fit indices were CFI = 0.890, RMSEA = 0.073.

**Table 3:** Confirmatory factor analysis of the factor models of previous studies with the second set of PBI data (n = 2330).

Study	Year	Country	No. of factors	Factors	Items	Paternal form		Maternal form	
						CFI	RMSEA	CFI	RMSEA
Kitamura et al.	1993	Japan	2	Care	1, 2, 4, 5, 6, 11, 12, 14, 16, 17, 18, 24	0.844	0.082	0.834	0.089
				Over-protection	3, 7, 8, 9, 10, 13, 15, 19, 20, 21, 22, 23, 25				
Uji et al.	2006	Japan	4	Care	1, 5, 6, 11, 12, 17	0.879	0.073	0.890	0.073
				Indifference	2, 4, 14, 16, 18, 24				
				Over-protection	8, 9, 10, 13, 19, 20, 23				
				Autonomy	3, 7, 15, 21, 22, 25				
Sato et al.	2021	Japan	3	Care	1, 2, 4, 5, 6, 11, 12, 16, 17, 18, 24	0.900	0.070	0.906	0.072
Current study	2022	Japan	3	Interference	8, 9, 10, 13, 19, 20				
				Autonomy	3, 15, 21, 22, 25				

### Analyses of multiple-group invariance

Table 4 shows the results of the analyses of multiple-group invariance. We found complete invariance (residual invariance) of the PBI ratings across primipara and multipara women for the paternal and maternal forms; this analysis included 4094 subjects (1955 primipara and 2139 multipara women) after the exclusion of 539 women with missing parity data. In contrast, we found weak invariance (metric invariance) of the PBI ratings across the paternal and maternal forms (n = 4633).

**Table 4:** Measurement invariance between primipara and multipara groups, and between the paternal and maternal PBI forms.

	CFI	ΔCFI
**Primiparous v.s. multiparous (n = 4094)**		
Configural invariance	0.901	
Metric invariance	0.901	0.000
Scalar invariance	0.898	0.003
Residual invariance	0.888	0.010
**Paternal v.s. maternal form (n = 4633)**		
Configural invariance	0.902	
Metric invariance	0.893	0.009
Scalar invariance	0.833	0.060

### Comparison of the mean value of each factor between the paternal and maternal forms

The factor 1 score was significantly higher in the maternal form than in the paternal form (23.97 ± 6.67 vs. 28.00 ± 5.54, p < 0.001) (Table 5). Higher factor 1 scores are considered to indicate better parenting styles with high “care”. The factor 2 score was significantly lower in the paternal form than in the maternal form (3.06 ± 2.52 vs. 3.45 ± 3.24, p < 0.001) (Table 5). Lower factor 2 scores are considered to indicate better parenting styles with less “interference”. The mean factor 3 score did not significantly differ between the paternal and maternal forms (p = 0.155) (Table 5).

**Table 5:** Comparison of the mean value of each factor between the paternal and maternal forms, and between primipara and multipara women.

	Paternal form			Maternal form			Paternal vs maternal form					
							Factor 1		Factor 2		Factor 3	
	Factor 1	Factor 2	Factor 3	Factor 1	Factor 2	Factor 3	t value	p value	t value	p value	t value	p value
Total women (n = 4633)	23.97 (6.67)	3.06 (2.52)	2.78 (2.76)	28.00 (5.54)	3.45 (3.24)	2.85 (2.85)	− 31.22	< 0.001*	− 7.080	< 0.001*	− 1.423	0.155
Primipara women (n = 1955)	24.34 (6.60)	3.06 (2.39)	2.54 (2.62)	28.42 (5.25)	3.60 (3.24)	2.68 (2.75)						
Multipara women (n = 2139)	23.64 (6.71)	3.06 (2.63)	3.00 (2.86)	27.61 (5.77)	3.32 (3.22)	3.00 (2.92)						
**Primipara vs multipara**												
t value	3.44	0.005	− 5.288	4.662	2.833	− 3.637						
p value	0.001*	0.996	< 0.001*	< 0.001*	0.005*	< 0.001*						

*The level of significance was set at p < 0.006 according to the Bonferroni correction of nine statistical tests.

### Comparison of the mean value of each factor between primipara and multipara women

The paternal factor 1 score was significantly higher for primipara than multipara women (24.34 ± 6.60 vs. 23.64 ± 6.71, p = 0.001) (Table 5). The mean paternal factor 2 score did not significantly differ between primipara and multipara women (p = 0.996) (Table 5). The paternal factor 3 score was significantly lower for primipara than multipara women (2.54 ± 2.62 vs. 3.00 ± 2.86, p < 0.001) (Table 5). Lower factor 3 scores are considered to indicate better parenting styles regarding an attitude that promotes “autonomy”. Compared with multipara women, primipara women had a significantly higher maternal factor 1 score (28.42 ± 5.25 vs. 27.61 ± 5.77, p = 0.001), significantly higher maternal factor 2 score (3.32 ± 3.22 vs. 3.60 ± 3.24, p = 0.005), and significantly lower maternal factor 3 score (2.68 ± 2.75 vs. 3.00 ± 2.92, p < 0.001) (Table 5).

## Discussion

The present study revealed that the three-factor structure was most plausible when using the PBI for perinatal women. This three-factor structure was completely consistent with that reported in a previous Japanese study using the PBI data of primipara women^19^. In this previous Japanese study, the three factors were “care”, “interference”, and “autonomy”^19^. The three-factorial structure that divides the control factor into two distinct factors was almost consistent with several previous studies of English-speaking and other Western populations. However, because the items that make up each of the three factors vary slightly among these studies, we performed the CFA using each model of the studies by Kendler^10^, Murphy et al.^11^, Gómez-Beneyto et al.^9^, and Cubis et al.^8^. The results showed that the model fit was worsened in the CFA using three-factor structures of these studies (Table S1) compared with the CFA of our study and the study by Sato et al.^19^. These findings suggest that it is appropriate to adopt the three-factor structure of our study and the study by Sato et al.^19^ in perinatal mental health studies.

Using the data of primipara and multipara women enabled us to analyze the extent to which the factor structure was comparable between primipara and multipara groups. We found complete invariance of the PBI ratings across primipara and multipara women for the paternal and maternal forms. In other words, each of the PBI factors induced by our study and the study by Sato et al.^19^ can be tested or construed across primipara and multipara women. This is a very important finding, because previous studies have shown that perceived parenting before adolescence influences perinatal anxiety and depression, and that parity is a confounding factor in these relationships^5^. Consequently, this three-factor structure that showed measurement invariance in perinatal women can be used to accurately determine how the perceived parenting before adolescence influences women’s mental health in the perinatal period.

We also analyzed the extent to which the factor structure was comparable between data of the paternal and maternal forms. Rather than complete invariance, we found weak invariance of the PBI ratings across the paternal and maternal forms. In contrast, Xu et al.^27^ found complete invariance across the paternal and maternal forms using the PBI data of 43-year-old survivors in the British 1946 birth cohort. This discrepancy in the invariance between our study and that of Xu et al.^27^ may be due to differences in population and/or the time in which the subjects were born. Therefore, further studies are needed to verify the measurement invariance across the paternal and maternal forms in various populations and generations. Until this measurement variance is clarified, we should be cautious about examining the factor structure of the PBI and how each factor of the PBI influences certain psychophysiological factors by combining data from the paternal and maternal forms.

In the present study, the participants tended to rate fathers as less caring and less overprotective than mothers, which is consistent with previous studies^11,27,28^. Compared with primipara women, multipara women tended to rate fathers and mothers as less caring and as having less attitude that promotes autonomy. Interestingly, we could find that these tendencies became more pronounced in not paternal but maternal form as the number of childbirth increased, when we divided the multipara group into three groups with 1, 2 and 3 or more childbirths and performed group comparisons (data not shown). This finding might suggest that a woman’s experience rearing their own child changes their perception of parenting style. However, this requires verification in further longitudinal studies of the same individuals, because the present study adopted a cross-sectional design and no other studies have examined the differences in the perception of perceived parenting between primipara and multipara women.

Our study has several limitations that deserve further discussion. First, we did not collect data of family-structure history from childhood to adulthood. Moreover, we did not deal with data of current marital status such as married, cohabitating, single, separated and divorced. So, we could not evaluate the measurement invariance among groups stratified with these data. Second, we found differences in perceptions on perceived parenting by own parents between primipara and multipara women. However, as this study adapted cross-sectional design, we could not know the causal relationship between the experience of childbirth and the perceptions on perceived parenting by own parents.

This study included a large number of perinatal women and analyzed the huge amount of PBI data. Furthermore, we precisely evaluated the measurement invariance between primipara and multipara groups, and between the paternal and maternal PBI forms. The three-factor structure evaluated in this study is considered to be useful to accurately determine how the perceived parenting style before adolescence influences women’s mental health in the perinatal period.

## Supplementary Information

Supplementary Table S1.

## Data Availability

All relevant data are provided in the paper. We are not able to make the underlying data available to readers, because we do not have permission from the participating institutions to do so.
